# Clinicopathological characteristics, molecular landscape, and biomarker landscape for predicting the efficacy of PD-1/PD-L1 inhibitors in Chinese population with mismatch repair deficient urothelial carcinoma: a real-world study

**DOI:** 10.3389/fimmu.2023.1269097

**Published:** 2023-11-06

**Authors:** Yu-Ting Ma, Fang Hua, Xiu-Ming Zhong, Ying-Jie Xue, Jia Li, Yi-Cong Nie, Xue-Dong Zhang, Ji-Wei Ma, Cun-Hu Lin, Hao-Zhuang Zhang, Wei He, Dan Sha, Miao-Qing Zhao, Zhi-Gang Yao

**Affiliations:** ^1^ Department of Pathology, Shandong Provincial Hospital Affiliated to Shandong First Medical University, Jinan, Shandong, China; ^2^ Department of Pathology, Shandong Cancer Hospital and Institute, Shandong First Medical University and Shandong Academy of Medical Sciences, Jinan, Shandong, China; ^3^ Department of Microbiology and Immunology, Tulane University, New Orleans, LA, United States; ^4^ Department of Pathology, Liaocheng People’s Hospital, Liaocheng, Shandong, China; ^5^ Department of Urology, Shandong Provincial Hospital Affiliated to Shandong First Medical University, Jinan, Shandong, China; ^6^ Department of Oncology, Shandong Provincial Hospital Affiliated to Shandong First Medical University, Jinan, Shandong, China; ^7^ Department of Pathology, Shandong Provincial Hospital, Cheeloo College of Medicine, Shandong University, Jinan, Shandong, China

**Keywords:** urothelial carcinoma, mismatch repair, Lynch syndrome, PD-1/PD-L1, immunotherapy

## Abstract

Urothelial carcinoma (UC) with deficient mismatch repair (dMMR) is a specific subtype of UC characterized by the loss of mismatch repair (MMR) proteins and its association with Lynch syndrome (LS). However, comprehensive real-world data on the incidence, clinicopathological characteristics, molecular landscape, and biomarker landscape for predicting the efficacy of PD-1/PD-L1 inhibitors in the Chinese patients with dMMR UC remains unknown. We analyzed 374 patients with bladder urothelial carcinoma (BUC) and 232 patients with upper tract urothelial carcinoma (UTUC) using tissue microarrays, immunohistochemistry, and targeted next-generation sequencing. Results showed the incidence of dMMR UC was higher in the upper urinary tract than in the bladder. Genomic analysis identified frequent mutations in *KMT2D* and *KMT2C* genes and LS was confirmed in 53.8% of dMMR UC cases. dMMR UC cases displayed microsatellite instability-high (MSI-H) (PCR method) in 91.7% and tumor mutational burden-high (TMB-H) in 40% of cases. The density of intratumoral CD8+ T cells correlated with better overall survival in dMMR UC patients. Positive PD-L1 expression was found in 20% cases, but some patients positively responded to immunotherapy despite negative PD-L1 expression. Our findings provide valuable insights into the characteristics of dMMR UC in the Chinese population and highlights the relevance of genetic testing and immunotherapy biomarkers for treatment decisions.

## Introduction

Urothelial carcinoma (UC) is a common malignancy of the urinary system, including bladder UC (BUC) and upper tract UC (UTUC) involving the ureters and renal pelvis. BUC represents the majority (90%–95%) of UC cases, while primary UTUC is relatively rare, accounting for 5% to 10% of cases (1). Platinum-based chemotherapy has conventionally been the standard postoperative systemic therapy for high-grade invasive UC over the past three decades. However, immune checkpoint inhibitors (ICIs) targeting programmed cell death 1 (PD-1) and PD-1 ligand (PD-L1) have gained prominence as vital treatments for advanced and metastatic UC (2, 3). Nonetheless, the response to PD-1/PD-L1 targeted ICIs therapy remains variable among UC patients (4), underscoring the need for predictive biomarkers that can identify individuals likely to respond positively to immunotherapy (5).

The mismatch repair (MMR) system plays a critical role in DNA repair by recognizing and correcting DNA mismatches during replication. Mutations or defects in MMR genes lead to deficient mismatch repair (dMMR) and genomic microsatellite instability (MSI). Lynch syndrome (LS) is an autosomal dominant hereditary tumor syndrome caused by germline pathogenic variants in MMR genes (MLH1, MSH2, MSH6, and PMS2) and/or EPCAM (6). LS is primarily associated with colorectal cancer (CRC) and endometrial cancer (EC), but it can also affect other organs such as the stomach, small bowel, prostate, brain, and skin. UTUC has also been recognized as a prevalent cancer in LS, with a lifetime risk ranging from 2.9% overall to 28% based on individual risk factors (7, 8). A retrospective analysis of databases revealed that a significant proportion (21.3%) of newly diagnosed UTUC patients might have LS as the underlying cause (9). This underestimation has significant implications for clinical practice, highlighting the importance of considering UC in the context of LS.

dMMR/LS-associated CRC often exhibits high levels of microsatellite instability (MSI-H), a high tumor mutation burden (TMB-H), and an abundant tumor-infiltrating lymphocytes (TILs) (10). These markers serve as predictive biomarkers for the efficacy of PD-1/PD-L1-targeted ICIs therapy. Notably, our previous study demonstrated significant benefits of PD-1 inhibitors in a patient with dMMR/LS-associated UC (11). However, the precise biomarker landscape for predicting the efficacy of PD-1/PD-L1 inhibitors in dMMR UC remains uncertain. In this study, we aim to select dMMR UC samples from BUC and UTUC using tissue microarrays (TMA) and MMR immunohistochemistry (IHC). We will investigate the clinicopathological characteristics, molecular landscape, and the expression of biomarkers such as MSI, TMB, TILs count, and others in dMMR UC. This investigation will contribute to predicting the efficacy of immunotherapy in UC.

## Materials and methods

### Patient cohort and tissue microarray construction

A comprehensive search was conducted in the database of Shandong Provincial Hospital Affiliated to Shandong First Medical University, Jinan, China, to identify all patients diagnosed with bladder urothelial carcinoma (BUC) and/or upper tract urothelial carcinoma (UTUC) who underwent surgical resection between 2018 and 2022. Patients who received neoadjuvant chemoradiotherapy were excluded from the study. Clinical data corresponding to the patients were obtained through a retrospective review of their medical records. Following pathological review, triplicate 1 mm diameter cores were sampled from epithelium-rich areas of formalin-fixed paraffin-embedded (FFPE) primary tumor tissue to construct a tissue microarray (TMA) using the TMA Master II instrument (3DHISTECH, Budapest, Hungary). Hematoxylin and eosin-stained (H&E) sections was examined from each TMA block to confirm the adequacy of tumor samples.

### Immunohistochemistry staining and assessment

Both microarray and whole section slides underwent the same staining technique. 5 μm thick sections of FFPE tissue were deparaffinized, rehydrated, and subjected to antigen retrieval by boiling in a sodium citrate buffer (pH 6.0) in a pressure cooker. Following antigen retrieval, the slides were blocked with 3% H_2_O_2_ and 5% goat serum, and the incubated with the primary antibody (MLH1: clone ES05, 1:100, PMS2: clone EP51, 1:50, MSH2: clone RED2, 1:100, and MSH6: clone EP49, 1:50, PD-L1: clone 22C3, 1:250, DAKO, Santa Clara, CA, USA; CD8: clone SP16, 1:200, CD4: PD-1: clone OTI4F10, 1:100, CD3, clone LN10, 1:100, ZSGB-BIO, Beijing, China). After washing with PBS, slides were incubated with biotinylated secondary antibody (ZSGB-BIO, Beijing, China), followed by ABC reagent (ZSGB-BIO, Beijing, China) and stable diaminobenzidine (ZSGB-BIO, Beijing, China). All sections were lightly counterstained with hematoxylin and mounted with aqueous mounting medium (ZSGB-BIO, Beijing, China). Negative controls were prepared using mouse or rabbit immunoglobulin-G at the same concentration as the primary antibodies.

The absence of detectable nuclear staining in neoplastic cells was considered indicative of deficient expression of MLH1, MSH2, MSH6, and PMS2. The adjacent non-neoplastic epithelium, stromal cells, or lymphocytes exhibiting intact nuclear staining were used as internal positive controls. CD3+ T cells, CD8+ T cells, and CD4+ T cells were quantified using ImageJ software (https://imagej.nih.gov/ij/index.html). Ten to twenty fields of view were counted at 40 ×, depending on the density of the CD8+ T cells within the tumor. The tumor proportion score (TPS) is determined as the percentage of PD-L1 positive stained tumor cells (TCs) with at least partial membrane staining relative to the total number of TCs, excluding tumor-associated interstitial cells, necrotic, normal, or non-neoplastic cells from the evaluation. The combined positive score (CPS) was defined as the number of PD-L1 or PD-1 positive cells (TCs, lymphocytes, and macrophages) divided by the total number of TCs multiplied by 100.

### Targeted NGS and sequence analysis

Genomic DNA was extracted from FFPE dMMR tumor tissues and corresponding normal tissues using the QIAamp DNA FFPE Tissue Kit (Qiagen, Shanghai, China), following the manufacturer’s instructions. The sheared genomic DNA fragments ranging from 200 to 400 bp were purified using the Agencourt AMPure XP Kit (Beckman Coulter, CA, USA). These fragments were then subjected to hybridization with capture probes baits, followed by selection using magnetic beads and amplification. Target capture was performed using a commercial OncoScreen Plus 520 panel (Burning Rock Biotech, Guangzhou, China), which covers genes related UC, tumor mutational burden (TMB), microsatellite instability (MSI), DNA damage response (DDR) pathway genes, as well as other factors relevant to positive and negative prediction and hyperprogression in immune and targeted therapies. The OncoScreen Plus 520 panel was developed through literature research, database analysis, clinical validation, and the selection of validated genes and biomarkers, and has been applied in gene testing studies for various types of cancer in both clinical and experimental settings (12, 13). Indexed libraries were subsequently sequenced on Illumina NextSeq 500 system (Illumina, Inc., USA) with paired-end reads and average sequencing depth of 1000×. The analysis of sequence data, along with variant verification, MSI status assessment, and TMB determination, followed previously described protocols (12, 13). The MSI status was detected with MSIsensor (V0.2), which was defined as the percentage of unstable microsatellite sites divided by total number of microsatellite sites surveyed. MSIsensor scores ≥ 10 and < 10 are defined as MSI-High (MSI-H) and Microsatellite Stable (MSS), respectively (14). TMB was defined as the number of all nonsynonymous mutations and indels per megabase of the genome examined. TMB > 10 Muts/Mb was defined as TMB-High (TMB-H). Tumor neoantigen burden (TNB) was measured as the number of mutations that could generate neoantigens per megabase (Neos/Mb). TNB > 4.5 Neos/Mb was defined as TNB-High (TNB-H) (15).

### Microsatellite instability analysis using PCR

To test the MSI status based on Polymerase Chain Reaction (PCR) and capillary electrophoresis resolution (MSI-PCR), DNA was extracted from FFPE tumor and normal tissue for each case. Sections were deparaffinized, rehydrated, and digested with proteinase K. From the lysate, the DNA was purified using the Wizard DNA Clean-Up System (Promega, Mannheim, Germany). For PCR, 25 ng of DNA were used per reaction. MSI testing was performed using the MSI Analysis System, Version 1.2 (Promega, Beijing, China), analyzing five nearly monomorphic mononucleotide markers (BAT-25, BAT-26, NR-21, NR-24, and MONO-27) for MSI determination and two polymorphic pentanucleotide markers (Penta C and Penta D) for sample identification. Negative, positive, and water (blank) controls were used. MSI-high (MSI-H) was defined as the presence of novel alleles in the tumor sample, but absence in the corresponding normal sample (indicating MSI) at two or more of the five mononucleotide repeat markers tested. MSI-low (MSI-L) was defined as any cases with peak shifts within one of the five regions tested. Microsatellite stable (MSS) was defined as no microsatellite instability detected at all five mononucleotide repeat markers tested.

### Statistical analysis

Statistical significance of differences between clinical and demographic variables was assessed using Pearson’s Chi-square test or Fisher’s exact test. The comparison of age between dMMR and pMMR groups was conducted using an independent samples t-test. The correlation between the two variables was evaluated using the Pearson correlation coefficient or Spearman’s rank correlation test. Survival analysis was performed using the Kaplan–Meier method with the log-rank test and the Cox proportional hazards model. Hazard ratios (HRs) and corresponding 95% confidence intervals (CIs) were estimated from the Cox analyses. A significance level of *P* < 0.05 was considered statistically significant. All analyses and graphs were conducted using SPSS Statistics 21 software (SPSS Inc., Chicago, IL, USA) and GraphPad Prism 9.5 software (San Diego, CA, USA).

## Results

### Clinicopathologic characteristics of dMMR UC

The cohort’s clinicopathological parameters are summarized in **Table 1**. We analyzed a total of 374 patients with BUC and 232 patients with UTUC in this study. Among them, we identified 15 cases of UC with simultaneous loss of MSH2 and MSH6 using TMA and IHC. These cases included 1 involved BUC, 9 involved UTUC, and 5 involved synchronous or metachronous occurrences of BUC and UTUC. The incidence of dMMR BUC within the BUC subgroup was only 1.6%. In the cohort, no cases with MLH1 and/or PMS2 loss were identified. dMMR BUC had a slightly lower average onset age (62.3 ± 16.7 vs. 65.4 ± 11.4) compared to proficient mismatch repair (pMMR) BUC. However, these differences were not statistically significant (*P* > 0.05). Furthermore, dMMR BUC showed an increased likelihood of tumor metastasis (M1 stage) compared to pMMR BUC (*P* < 0.05). There were no significant differences observed between dMMR and pMMR BUC in terms of patient gender, tumor grade, T stage, or N stage (*P* > 0.05). Among the UTUC cases, dMMR UTUC accounted for 6% of the total cases. Similar to dMMR BUC, dMMR UTUC had a slightly lower average age of onset (64.0 ± 13.5 *vs.* 65.7 ± 9.9) compared to pMMR UTUC. However, these differences were not statistically significant (*P* > 0.05). Unlike pMMR UTUC, dMMR UTUC more frequently presented as low-grade tumors (50% *vs.* 84.9%) and predominantly fell within the Ta-T1 stage (78.6% *vs.* 29.8%, **Tables 1** and **2**). Additionally, we observed that dMMR BUC and dMMR UTUC often co-occurred synchronously or metachronously (33.3%). Furthermore, patients with dMMR BUC and UTUC exhibited a significantly higher rate of personal and family histories of cancer than pMMR UC (*P* < 0.001) (**Table 1**).

**Table 1:** Patient demographics and clinicopathologic characteristics of pMMR and dMMR UC.

Characteristics	Bladder UC				UTUC			
	Overall Cohort N (%)	pMMR N (%)	dMMR N (%)	*P* value	Overall Cohort N (%)	pMMR N (%)	dMMR N (%)	*P* value
**Age (Mean ± SD)**	65.4 ± 11.4	65.4 ± 11.4	62.3 ± 16.7	>0.05	65.6 ± 10.1	65.7 ± 9.9	64.0 ± 13.5	>0.05
**Gender**				>0.05				>0.05
Male	312 (83.4)	309 (82.6)	3 (0.8)		133 (57.3)	126 (54.3)	7 (3.0)	
Female	62 (16.6)	59 (15.8)	3 (0.8)		99 (42.7)	92 (39.7)	7 (3.0)	
**Location**				**<0.001**				
Bladder	361 (96.5)	360 (96.3)	1 (0.3)					
Bladder + UTUC	13 (3.5)	8 (2.1)	5 (1.3)		13 (5.6)	8 (3.4)	5 (2.2)	**<0.001**
UTUC					219 (94.4)	210 (90.5)	9 (3.9)	
**Grade**				>0.05				**<0.01**
High	265 (70.9)	262 (70.1)	3 (0.8)		192 (82.8)	185 (79.7)	7 (3.0)	
Low	109 (29.1)	106 (28.3)	3 (0.8)		40 (17.2)	33 (14.2)	7 (3.0)	
**T stage**				>0.05				**<0.01**
Ta - T1	262 (70.1)	257 (68.7)	5(1.3)		76 (42.7)	65 (36.5)	11 (6.2)	
T2 - T4	112 (29.9)	111 (29.7)	1 (0.3)		102 (57.3)	153 (86.0)	3 (1.7)	
**N stage**				>0.05				>0.05
N0	362 (96.8)	356 (95.2)	6 (1.6)		215 (92.7)	201 (86.6)	14 (6.0)	
N1-N2	12 (3.2)	12 (3.2)	0 (0.0)		17 (7.3)	17 (7.3)	0 (0.0)	
**M stage**				**<0.05**				>0.05
M0	360 (96.3)	356 (95.2)	4 (1.1)		210 (90.5)	198 (85.3)	12 (5.2)	
M1	14 (3.7)	12 (3.2)	2 (0.5)		22 (9.5)	20 (8.6)	2 (0.9)	
**Personal cancer history**				**<0.001**				**<0.001**
Yes	30 (8.0)	25 (6.7)	5 (1.3)		21 (9.1)	11 (4.7)	10 (4.3)	
No	344 (92.0)	343 (91.7)	1 (0.3)		211 (90.9)	207 (89.2)	4 (1.7)	
**Family cancer history**				**<0.001**				**<0.001**
Yes	11 (2.9)	6 (1.6)	5 (1.3)		15 (6.5)	6 (2.6)	9 (3.9)	
No	363 (97.1)	162 (43.3)	1 (0.3)		217 (93.5)	212 (91.4)	5 (2.2)	

**Table 2:** Clinicopathologic features and gene testing of the cases with dMMR UC.

Case	Age	Sex	Location	Grade	TNM stage	Personal cancer history	Family cancer history	Germline mutation	Mutation Type	Clinical Significance
1	72	Female	Left ureter	low	T1N0M0	Yes	Yes	MSH2 c.2006-2A>G	Splicing variant	Pathogenic
2	61	Male	Left renal pelvis	High	T2N0M0	Yes	No	MSH2 c.1216C>T: p.Arg406*	Nonsense	Pathogenic
3	64	Male	Left and right ureter	High	T1N0M0	Yes	No	MSH2 c.1165C>T: p.Arg389*	Nonsense	Pathogenic
4	69	Female	Right renal pelvis	High	T3N0M0	Yes	Yes	MSH2 c.643C>T: p.Gln215*	Nonsense	Pathogenic
5	45	Female	Left ureter and bladder	Low	T1N0M0	Yes	Yes	MSH2 c.942 + 3A>T	Splicing variant	Pathogenic
6	55	Male	Left ureter	High	T1N0M0	No	Yes	MSH2 c.1477C>T: p.Gln493* MSH2 c.1861C>T: p.Arg621*	Nonsense Nonsense	Pathogenic Pathogenic
7	61	Female	Right ureter and bladder	Low	T1N0M0	Yes	Yes	No data		
8	76	Male	Bladder	High	T1N0M0	No	No	No data		
9	87	Male	Right ureter and bladder	Low	T1N0M0	Yes	Yes	Not detected		
10	55	Female	Left ureter	Low	TaN0M0	No	No	Not detected		
11	69	Female	Left ureter	Low	T1N0M0	Yes	Yes	Not detected		
12	59	Male	Right ureter	Low	T1N0M0	No	No	Not detected		
13	92	Male	Left ureter	High	T1N0M0	No	No	Not detected		
14	45	Female	Right ureter and bladder	High	T1N0M1	Yes	Yes	Not detected		
15	62	Male	Right ureter and bladder	High	T3N0M1	Yes	Yes	MSH2 c.715C>T: p.Gln239*	Nonsense	Pathogenic

### Histopathologic characteristics of dMMR UC

**Figure 1:**
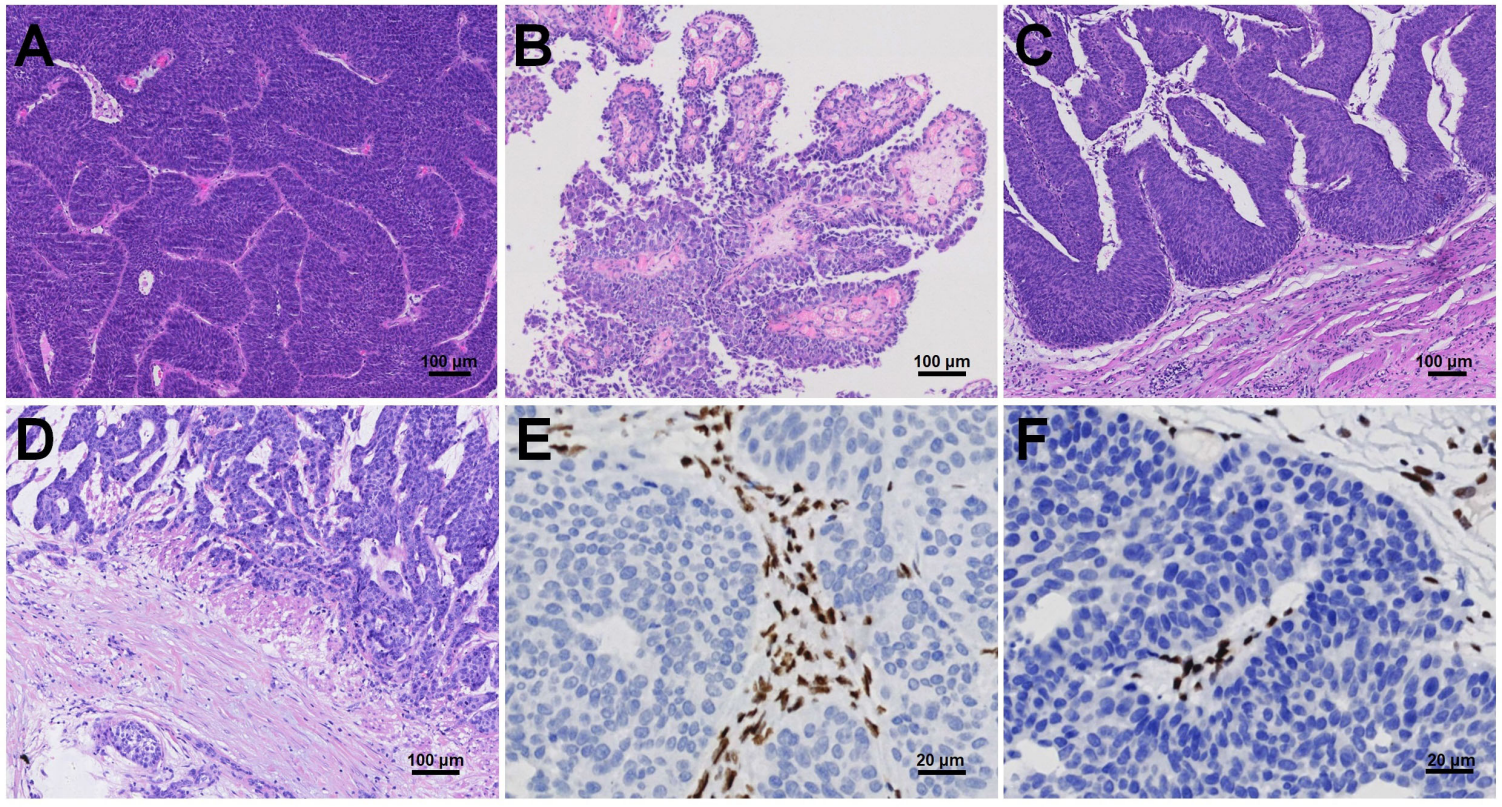
Histopathologic characteristics of dMMR UC, including inverted **(A)** or papillary **(B)** growth patterns with either pushing borders **(C)** or destructive infiltrative edges **(D)**. Immunohistochemical staining revealed loss of MSH2 **(E)** and MSH6 **(F)**.

We evaluated the histopathologic characteristics of dMMR UC by reviewing all H&E sections. Inverted growth was observed in all 15 (100%) cases, with 10 cases exhibiting purely inverted characteristics (**Figure 1A**) and 5 cases displaying a mixed morphology characterized by focal inverted and papillary patterns (**Figure 1B**). Among the total cases, 8 (53.3%) cases were classified as high grade. 14 (93.3%) cases demonstrated pushing borders (**Figure 1C**), while only 1 (1.7%) case exhibited destructive infiltrative edges (**Figure 1D**). Immunohistochemical staining in a representative dMMR case revealed loss of MSH2 (**Figure 1E**) and MSH6 (**Figure 1F**).

### Somatic mutational landscape of dMMR UC

**Figure 2:**
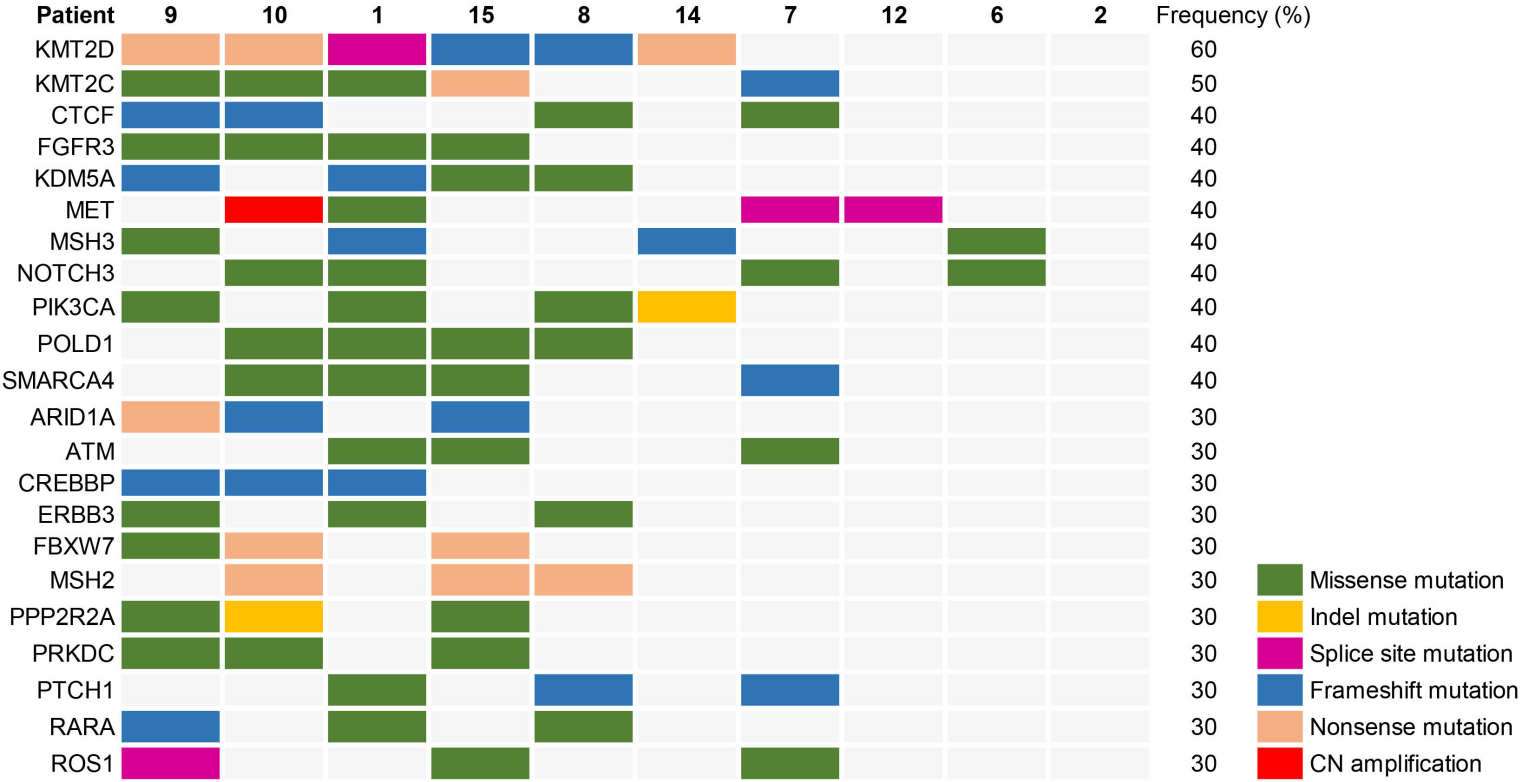
Somatic mutational landscape in 10 cases of dMMR UC.

According to patient informed consent, NGS was conducted on tumor tissues and corresponding normal tissues from 10 cases to investigate the somatic mutational landscape of dMMR UC. The most frequently mutated gene in dMMR UC was KMT2D (60%), with 3 (50%) cases exhibiting nonsense mutations, 2 (33.3%) cases showing frameshift mutations, and 1 (16.7%) case displaying a splice sites mutation (**Figure 2**). Notably, Patient 8 and 15 harbored the same frameshift mutation site (KMT2D c.3704del, p.G1235fs), while Patient 9 and 10 carried the same nonsense mutation site (KMT2D, c.5707C>T, p.R1903*). The second most frequently mutated gene in dMMR UC was KMT2C (50%), while 3 (60%) cases showing missense mutation, 1 (20%) case exhibiting a nonsense mutation, and 1 (20%) case displaying a frameshift mutation. Other notable variants included FGFR3 (40%), CTCF (40%), KDM5A (40%), MET (40%), MSH3 (40%), NOTCH3 (40%), PIK3CA (40%), POLD1 (40%), and SMARCA4 (40%) (**Figure 2**). Moreover, Patient 1, 9, 10 and 15 carried the same missense mutation site of FGFR3 (c.742C>T, p.R248C) (40%), with two of them diagnosed as LS, and the other two without MSH2 germline mutations. Less frequent mutations in dMMR UC included ARD1A (30%), ATM (30%), CREBBP (30%), FBXW7 (30%), ROS1 (30%), and others (**Figure 2**).

### Germline variants testing

**Figure 3:**
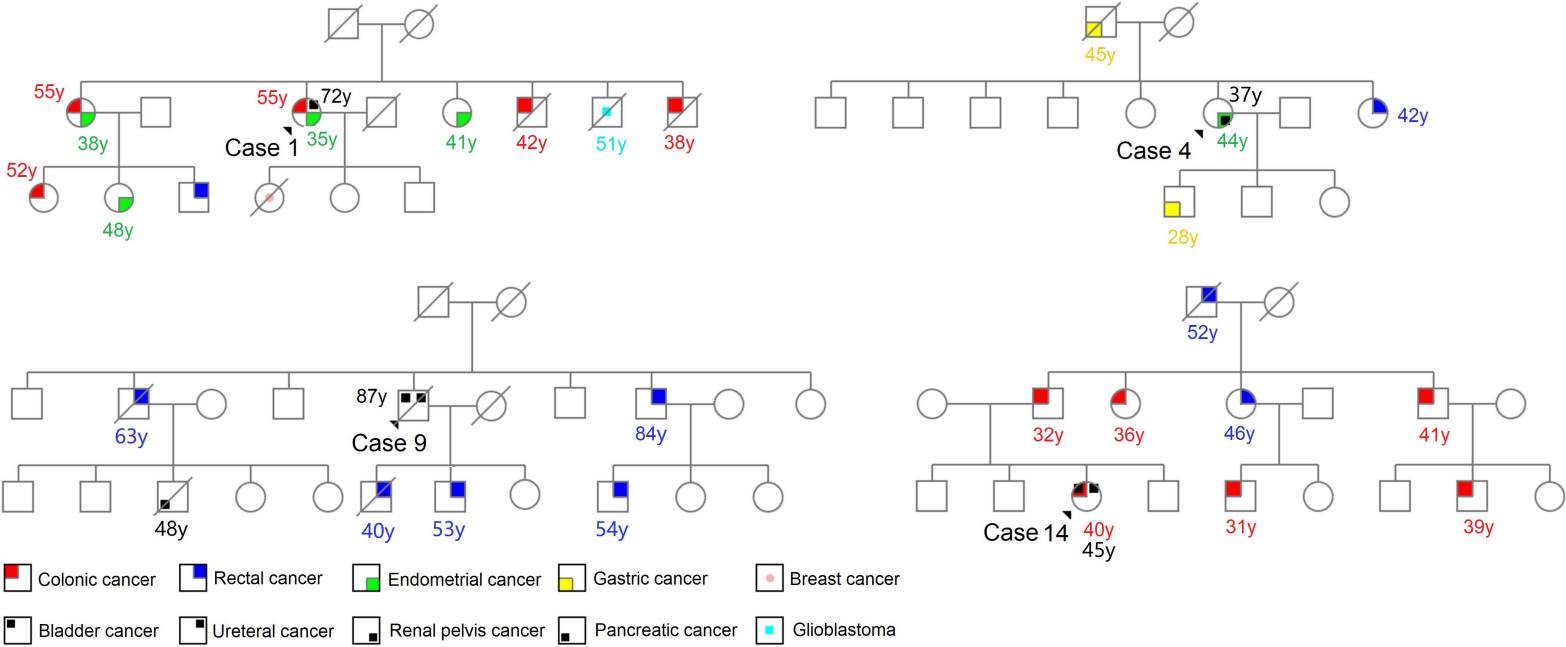
Representative pedigrees of Lynch syndrome.

In the clinical assessment, 10 (66.7%) out of 15 UC with dMMR had personal histories of Lynch syndrome (LS)-associated neoplasms, such as CRC and EC. Additionally, 9 (60%) cases had family histories of these neoplasms. Six (40%) cases satisfied the Amsterdam II criteria, while 10 (66.7%) cases fulfilled the revised Bethesda guidelines, both indicating a suspicion of LS. **Figure 3** displays the representative pedigrees. To identify potential germline variants associated with LS, germline MLH1, PMS2, MSH2, MSH6, and EPCAM genes were tested using NGS in 13 cases of dMMR UC based on patient informed consent. The analysis revealed pathogenic mutations in the MSH2 gene in 7 (53.8%) cases, including 5 cases with nonsense mutations and 2 cases with splice variants (**Table 2**).

### MSI, TMB, and TNB analysis

Microsatellite Instability (MSI) status was assessed in dMMR UC using two different methods: NGS (MSI-NGS) and the MSI Promega Analysis System (MSI-PCR). The results are summarized in **Supplemental Table S1**. MSI-H was observed in 8 (53.3%) cases using the MSI-NGS method, while MSI-L and MSS were found in 0 and 7 (46.7%) cases, respectively. With the MSI-PCR method, MSI-H was detected in 11 (91.7%) cases, MSI-L in 1 (8.3%) case, and none of the 12 cases showed MSS (**Supplemental Figure S1**). Among the analyzed cases, instability was observed in 5 mononucleotide markers: BAT-25, MONO-27, NR-21, BAT-26, and NR-24, with frequencies of 75%, 66.7%, 66.7%, 58.3%, and 41.7%, respectively. A total of 12 dMMR UC samples underwent MSI testing using both methods, with an overall concordance rate of 41.7% (5 out of 12 cases). Among the cases identified as MSS by the NGS method, 6 out of 7 cases (85.7%) were shown to be MSI-H using the MSI-PCR method, indicating a sensitivity of 45.5% for the NGS method. The concordance rate for MSI-H status assessed using NGS and dMMR evaluated by IHC was 41.7%. Notably, the concordance rate for MSI-H status between the MSI-PCR testing and dMMR evaluation by IHC was 91.7%, indicating a high level of consistency.

TMB analysis was conducted using NGS. Among the 15 cases examined, 6 (40%) displayed high TMB (TMB-H) with a range of 29.91 to 68.79 mutations per megabase (mut/Mb), and a mean of 44.53 mut/Mb (**Supplemental Table S1**). TMB exhibited a strong positive correlation with the number of gene mutations (R^2^ = 0.9830, *P* < 0.01, **Supplemental Figure S2A**). Out of the 11 cases analyzed, there was a concordance rate of 4 (36.4%) between the identification of MSI-H by PCR and TMB-H. Furthermore, TMB displayed a positive correlation with the proportion of unstable microsatellite loci detected by NGS (R^2^ = 0.6208, *P* < 0.01, **Supplemental Figure S2B**).

TNB predictions were conducted for patients with dMMR UC. Among the examined cases, 6 (40%) exhibited a high TNB (> 4.5 Neos/Mb), ranging from 20 to 90 Neos/Mb, with a mean of 55.17 mut/Mb. As expected, TNB demonstrated a significant positive correlation with the number of gene mutations (R^2^ = 0.5593, *P* < 0.05, **Supplemental Figure S2C**), the proportion of unstable microsatellite loci detected by NGS (R^2^ = 0.6208, *P* < 0.01, **Supplemental Figure S2D**), and TMB (R^2^ = 0.6460, *P* < 0.01, **Supplemental Figure S2E**).

### PD-1/PD-L1 and tumor-infiltrating T cells analysis

**Figure 4:**
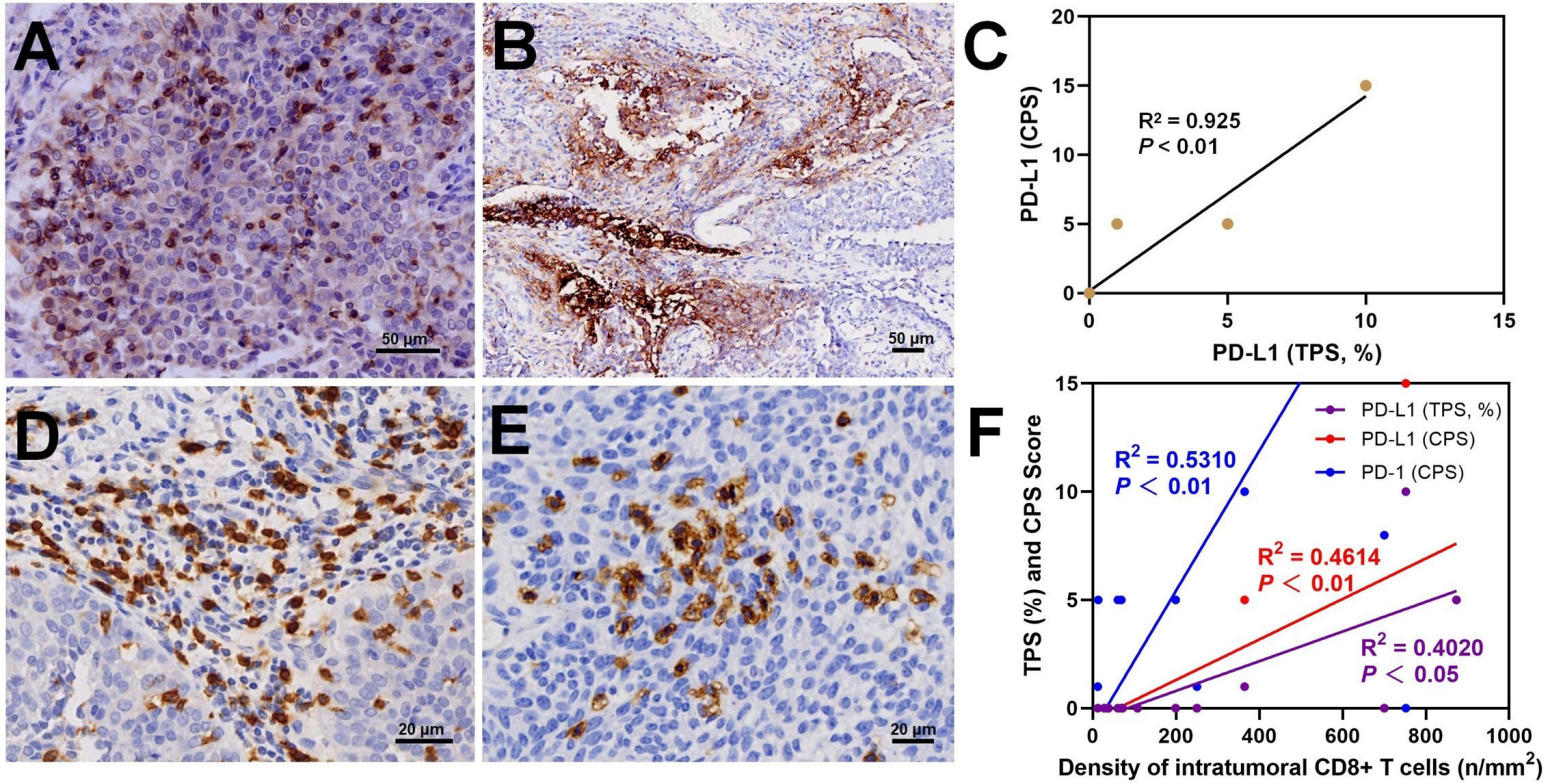
In dMMR UC, immunostaining results showed intratumoral PD-1+ lymphocytes **(A)**, PD-L1+ tumor cells **(B)**, a positive correlation between TPS score and CPS score **(C)**, and peritumoral **(D)** and intratumoral **(E)** CD8+ T lymphocytes with a positive correlation between the density of intratumoral CD8+ cells and PD-1 and PD-L1 **(F)**.

Morphologically, PD-1 immunostaining was observed on the membrane of immune cells (ICs) with CPS scores ranging from 1 to 60 (**Supplemental Table S1**). Positive PD-1 expression was found in 9 (60%) cases. Notably, a significant number of intratumoral ICs exhibited intense positive staining of PD-1 (**Figure 4A**). Positive PD-L1 immunostaining was observed on the cell membrane of tumor cells (**Figure 4B**). Based on the TPS and CPS scores for PD-L1 expression, three (20%) cases had TPS ≥ 1% and CPS ≥ 1, while twelve (80%) cases had negative PD-L1 status. The TPS scores of cases with positive PD-L1 expression ranged from 1% to 5%, while the CPS scores ranged from 5 to 15. A strong positive correlation was observed between the TPS and CPS scores of PD-L1 staining (R^2^ = 0.925, P < 0.01, **Figure 4C**). However, no correlation was found between PD-1 and PD-L1 expression (*P* > 0.05).

Peritumoral CD3+ T cell infiltration was observed in 15 cases with dMMR UC, with a density ranging from 724.01 to 4070.54/mm^2^ (**Supplemental Table S2**). In 80% of the cases, the density of peritumoral CD3+ T cells was > 2000/mm^2^. The density of peritumoral CD8+ T cells ranged from 2347.27 to 84.62/mm^2^, with 6 (40%) cases exceeding 800/mm^2^ (**Figure 4D**). The percentage of peritumoral CD8+ T cells among CD3+ T cells ranged from 10.58% to 65.05%. In 5 (33.3%) cases, the percentage of peritumoral CD8+ T cell was ≥30%. A significant positive correlation was observed between peritumoral CD8+ and CD3+ T cell density (R^2^ = 0.4590, *P* < 0.01). The density of intratumoral CD8+ T cells ranged from 12.09 to 873.60/mm^2^ (**Supplemental Table S2**). In 7 (46.7%) cases, the intratumoral CD8+ T cell density exceeded 100/mm^2^. Morphologically, these cases show abundant CD8+ T cells distributed in patches or clusters within the tumor cell nests (**Figure 4E**). The density of peritumoral and intratumoral CD8+ T cells displayed a positive correlation (R^2^ = 0.6091, *P* < 0.01, **Supplemental Figure S2F**). Moreover, positive correlations were also found between intratumoral CD8+ T cells density and PD-L1 score (TPS, R^2^ = 0.531, *P* < 0.01), PD-L1 score (CPS, R^2^ = 0.4614, *P* < 0.01), and PD-1 score (CPS, R^2^ = 0.402, *P* < 0.05) (**Figure 4F**). No correlation was found between the intratumoral CD8+ T cell percentage and TNB (R^2^ = 0.1246, *P* > 0.05). The evaluation of CD4+ T cell content showed that in 6 (40%) cases, the peritumoral CD4/CD3 ratio was > 50%.

### Immunotherapy associated somatic variants analysis

**Figure 5:**
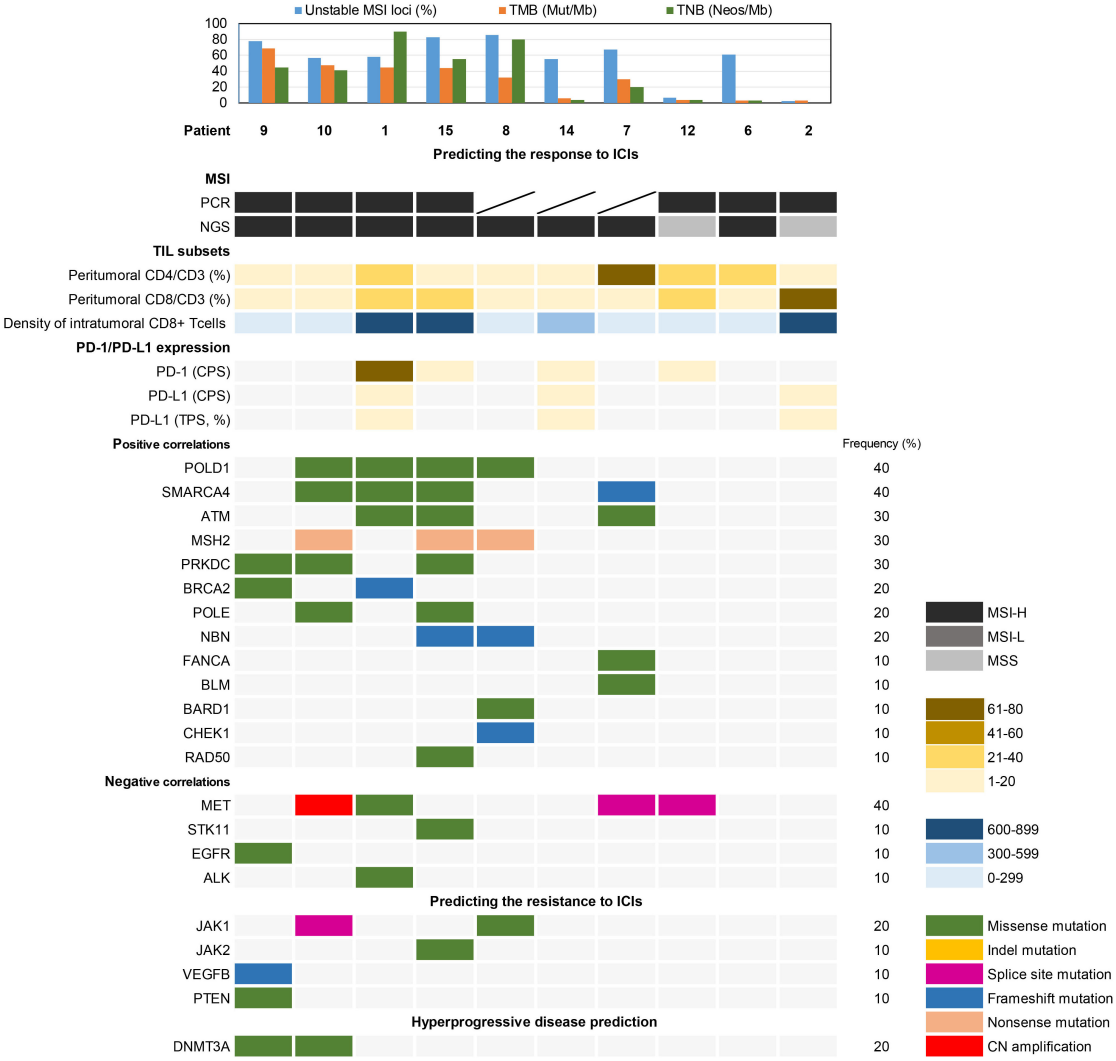
Biomarker analysis of somatic variations associated with the prediction of immunotherapy response.

NGS analysis revealed variants in 20 genes related to the DDR pathway (**Figure 5**). *POLD1* and *SMARCA4*, positively correlated biomarkers for predicting the efficacy of immune checkpoint inhibitors (ICIs), exhibited variations in 4 (40%) cases each. Additionally, *ATM*, *MSH2*, and *PRKDC* mutations were detected in 3 (30%) cases each. On the other hand, the negatively correlated biomarker *MET* showed variants in 4 cases (40%), with only Patient 1 having a missense mutation (c.3979C>T, p.R1327C) within the tyrosine kinase domain. Patient 1, 9, and 15 exhibited missense mutations in *ALK*, *EGFR*, and *STK11*, respectively. Resistance biomarker analysis for immunotherapy identified *JAK1* mutations in Patient 8 and 10, *JAK2* and *VEGFB*/*PTEN* mutations were found in Patient 9 and 15, respectively. Additionally, *DNMT3A* missense mutations were observed in Patient 9 and 10, associated with hyperprogressive disease.

### Prognostic analysis

**Figure 6:**
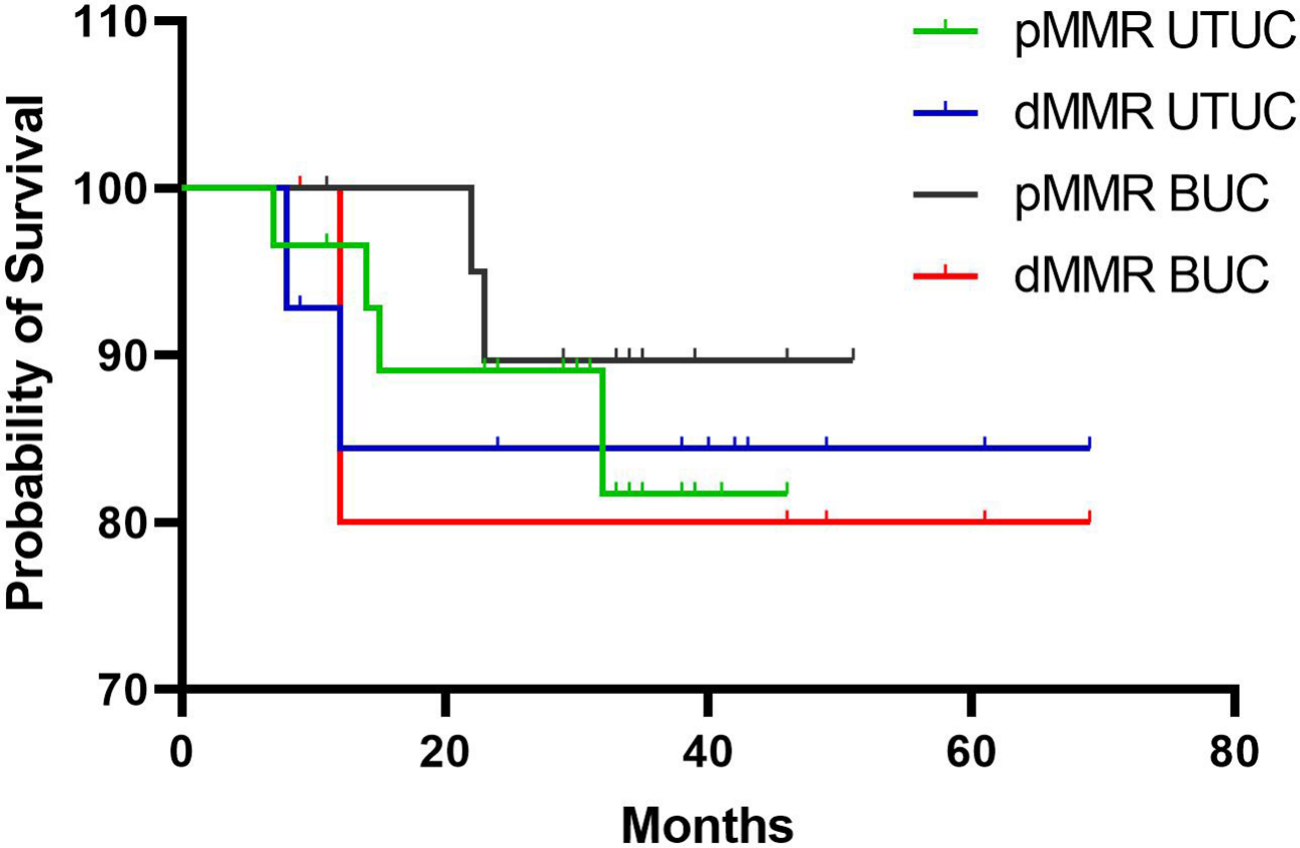
Kaplan-Meier analysis of overall survival rates in patients with dMMR and pMMR UC. BUC, bladder urothelial carcinoma; UTUC, upper tract urothelial carcinoma.

In this study, the median follow-up duration for all patients was 31.5 months (range: 7-69 months). While patients with dMMR BUC (41.0 *vs.* 31.3 months) and dMMR UTUC (32.6 *vs.* 28.7 months) showed higher average overall survival (OS) compared to their pMMR counterparts. Kaplan-Meier analyses revealed no significant differences in OS between pMMR and dMMR BUC (*P* > 0.05) or pMMR and dMMR UTUC (*P* > 0.05) (**Figure 6**). To investigate the association between clinicopathological characteristics and immunotherapy biomarkers, including age, tumor location, tumor grade, TNM stage, MSI (PCR), TMB values, intrastromal and intratumoral CD8+ T cells density, PD-L1 score (CPS), and OS, univariate Cox regression analysis was performed. The results showed that only the density of intratumoral CD8+ T cells exhibited a significant association with OS in our cohort (*P* < 0.05, HR = 0.170, 95% CI: 0.034-0.844).

### Representative cases

Patient 3, diagnosed with LS, initially underwent radical nephroureterectomy for right UTUC. Two and a half years later, the patient underwent left ureteral resection for upper and lower ureteral UC, along with nephrostomy. The patient subsequently received treatment with Tirelizumab for 5 cycles. Unfortunately, the patient died of systemic sepsis caused by the nephrostomy.

**Figure 7:**
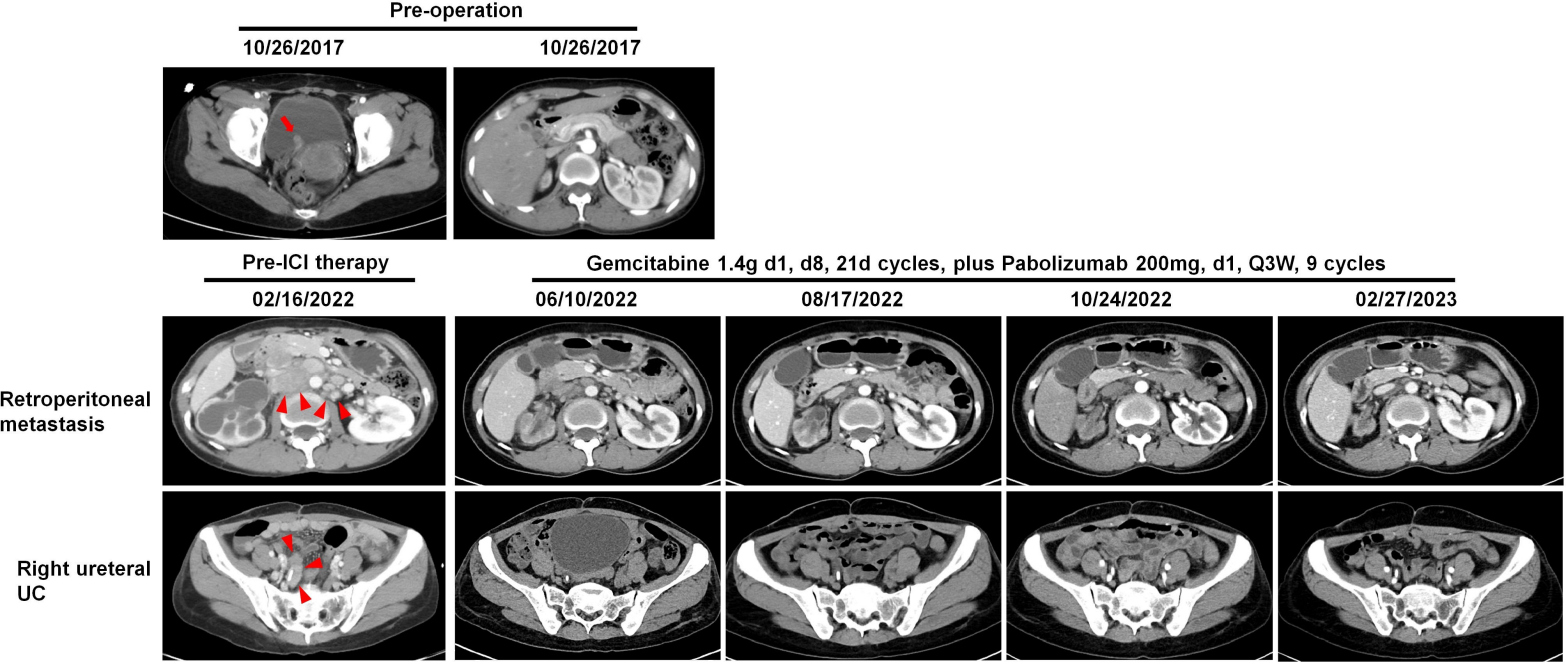
Patient 14: Images of preoperative bladder UC (red arrow), the following retroperitoneal metastatic lesions (red arrowheads), and right ureteral UC (red arrowheads) before and after combined immunotherapy, illustrating tumor regression and stabilization.

Two cases of metastatic dMMR UC (Patient 14 and 15) achieved complete remission following combination treatment with PD-1 inhibitors and chemotherapy. As our previous reports^11^, Patient 15 had concurrent ureteral and bladder UC, initially treated with nephroureterectomy and transurethral resection of bladder tumor. Despite disease progression with local bladder recurrence and lung/bone metastasis after cisplatin-based first-line chemotherapy and docetaxel second-line monotherapy, the patient achieved sustained response after five cycles of PD-1 inhibitor sintilimab (200 mg on day 1) combined with docetaxel (120 mg on day 2, every 21 days) over 31 months, showing disappearance of nodules in the bladder, lung, and left proximal femur. Patient 14 underwent partial cystectomy and right ureter reimplantation for BUC. Four years later, imaging revealed retroperitoneal metastasis and right ureteral UC (**Figure 7**). After 8 cycles of PD-1 inhibitor pembrolizumab (200 mg on day 1) combined with gemcitabine (1.4 g on day 1, every 8 days) therapy, both the retroperitoneal metastatic lesions and ureteral tumor significantly decreased in size. In terms of treatment side effects, Patient 15 experienced sintilimab-associated skin allergies, hypophysitis, and hypothyroidism, which improved with treatment^11^. Patient 14 experienced vomiting and severe neutropenia, which normalized after receiving granulocyte colony-stimulating factor. Thus, the side effects of immunotherapy in dMMR UC were manageable.

## Discussion

In this study, we investigated the clinicopathologic characteristics of dMMR UC. The incidence of dMMR BUC was found to be 1.6%, while that of dMMR UTUC was 6%. These findings align with previous studies, which have reported dMMR rates of 2% in muscle invasive bladder cancer (MIBC) cases and 5% to 11.3% in UTUC cases (8, 16). A recent Chinese cohort study on UTUC reported a 10.9% rate of MMR loss (17). Therefore, the upper urinary tract was the most common location for dMMR loss. Consistently, we observed that dMMR was consistently associated with combined loss of MSH2 and MSH6, and 53.8% of cases confirmed the presence of pathogenic germline variants in the MSH2 gene, supporting the diagnosis of Lynch syndrome (LS). Individuals with LS have a lifetime risk of developing UTUC ranging from 2.9% to 28%, which is 22 times higher than that of the general population (8). LS-associated UTUC cases often exhibit germline mutations in the MSH2 gene, with rates ranging from 63% to 100%, involving 77% of ureteral cancers and 74% of renal pelvis cancers (18). LS-associated BUC also shows a high germline mutation rate in *MSH2*, ranging from 69% to 79% (14, 19). LS-associated UTUC tends to occur at a younger age (62 years *vs.* 70 years), predominantly affects females (approximately 59%), and is commonly found in the ureter. Furthermore, 51% of LS-associated UTUC cases have a risk of metachronous bilateral ureteral cancer (18). Therefore, kidney-preserving strategies, such as ureteroscopic laser ablation or a neoadjuvant chemotherapy combination with immunotherapy, can be considered to preserve renal function and improve the quality of life and overall survival of patients (20). On the other hand, our study revealed some differences compared to previous studies. We found that dMMR UC was more likely to present as synchronous or metachronous ureteral and bladder UC, emphasizing the importance of thorough examination of the urinary system to exclude concurrent urothelial cancers. Additionally, dMMR UC was more frequently associated with personal tumor history and family history compared to pMMR UC, indicating the need for further germline testing to identify LS. This will enable family members to benefit from genetic counseling, as well as screening or surveillance protocols. Furthermore, dMMR UC typically exhibits an inverted growth pattern and is often diagnosed at pTa or pT1 stages (21, 22). These findings are consistent with our study results, and the combination of clinical data and histopathological characteristics may help identify patients at high risk of MMR protein expression loss.

In our study, we also investigated the molecular changes in dMMR UC. The mutational landscape revealed higher frequencies of alterations in *KMT2D* (60%), *KMT2C* (50%), *FGFR3* (40%), *KDM5A* (40%), and *SMARCA4* (40%). A previous study on LS-related UTUC confirmed the most common gene mutations as *KMT2D* (94%), *CREBBP* (82%), *ARID1A* (76%), and *SMARCA4* (76%) (14). In pMMR UTUC, the most frequently affected genes included the *TERT* promoter (49%), *KMT2D* (46%), *CDKN2A* (45%), *FGFR3* (45%), and *TP53* (35%) (23). This suggests that chromatin remodeling abnormalities may play crucial roles in the development of both dMMR and pMMR UC. Notably, Donahu et al. confirmed that *FGFR3* R248C hotspot mutation was highly enriched in LS associated UTUC (14). We also identified *FGFR3* R248C hotspot mutation in four cases of dMMR UC. This mutation leads to increased FGFR3 dimer stability and constitutive receptor activation, implicating the involvement of the phosphatidylinositol 3-kinase–AKT and mitogen-activated protein kinase signaling pathways in the pathogenesis of dMMR UC (24).

In addition, we evaluated the expression of biomarkers associated with the prediction of immunotherapy response, with a focus on MSI status in dMMR UC. Previous reports have shown that MSI occurs in a higher proportion of UTUC (3.9% to 28.1%) compared to BUC (<1%) (25, 26). Our findings indicated a concordance rate of 91.7% between dMMR and MSI-H (PCR method). However, the concordance rate between NGS and PCR for MSI detection was only 41.7%. This highlights the higher detection rate of MSI-H with PCR compared to NGS. Nevertheless, NGS analysis of MSI in UC has great potential due to its ability to analyze a larger number of microsatellite regions compared to PCR. Furthermore, we found a strong positive correlation and an 86.7% overlap between MSI-NGS, TMB, and TNB. The presence of dMMR/MSI-H/TMB-H in dMMR UC leads to a higher TNB, resulting in an increased number of neoantigens, an inflammatory microenvironment, and improved responses to ICIs therapy (27, 28).

The assessment of PD-L1 expression plays a crucial role in predicting the response to ICIs treatment. In a previous cohort study, it was found that 75% (three out of four) of high-grade urothelial carcinoma (HGUC) cases with dMMR had positive PD-L1 expression, whereas intact MMR HGUC cases showed a lower rate of 20% (16). The KEYNOTE-045 and KEYNOTE-052 studies demonstrated the superiority of pembrolizumab over chemotherapy in terms of longer overall survival and lower rates of treatment-related adverse events in advanced urothelial carcinoma (UC) patients with PD-L1 combined positive score (CPS) ≥ 10% as first- and second-line therapies (2, 3). However, it is important to note that not all PD-L1-positive patients respond significantly, and some PD-L1-negative patients still show a positive response to treatment (4). In our study, we observed PD-L1 positivity in only 20% of dMMR UC cases, and there was a positive correlation between CPS and tumor proportion score (TPS) for PD-L1 expression. Significant clinical benefits were observed in Patient 14 with low PD-L1 expression and Patient 15 with negative PD-L1 expression after treatment with PD-1 inhibitors. Another study involving Atezolizumab did not establish a significant correlation between PD-L1 expression and treatment outcome in either immune cells or tumor cells (29). This lack of correlation may be attributed to the dynamic interactions between cancer cells and their immune microenvironment, which lead to fluctuations in the immunological profile, including PD-L1 expression, TMB, and TILs (30). Hence, the use of clinically validated assays and PD-L1 scoring algorithms in UC, along with the integration of PD-L1 with other biomarkers, is crucial for accurately predicting the clinical response to ICIs.

We also observed abundant intratumoral CD8+ T cells in dMMR UC cases. Notably, even in Patient 14 and 15, which exhibited low or no PD-L1 expression, the presence of patchy or clustered CD8+ T cells was observed, and significant therapeutic benefits were obtained through PD-1 inhibitor therapy (11). A higher density of intratumoral CD8+ T cells is significantly associated with a greater rate of pathologic complete response in MIBC and metastatic BUC, regardless of PD-L1 expression, TMB, or DNA damage repair (DDR) gene mutations (29, 31, 32). Furthermore, a high density of intratumoral CD8+ T cell is associated with improved overall survival and disease-specific survival rates (33).

In our study, we examined somatic variants of biomarkers associated with immune therapy response, including positive or negative associations, as well as indications of immune resistance or hyperprogression. These biomarkers were not exclusively present in dMMR UC, suggesting their potential relevance for immune therapy in individual cases. For instance, the DDR pathway plays a critical role in maintaining genomic stability and repairing DNA damage. In non-MIBC, deleterious variants of DDR genes were more frequently detected in high-grade disease compared to low-grade disease (28.7% *vs.* 7.1%) (34). DDR deficiencies lead to DNA damage accumulation and increased immunogenicity in MIBC (35). In our study, we identified mutations in *POLD1* (40%), *SMARCA4* (40%), *ATM* (30%), *MSH2* (30%), and *PRKDC* (30%) in dMMR UC. However, we did not find a relationship between DDR mutations and tumor infiltrating CD8+ T cells, suggesting that DDR gene variations may need to be combined with other predictive markers.

We acknowledge the limitations of our study. We had a limited number of dMMR UC cases that received immunotherapy. The application of ICIs in the treatment of UC is selective, specifically targeting patients with a high risk of recurrence and those with advanced and metastatic disease (2, 3). In our study, 73.3% of the dMMR UC cases were classified as Ta/T1N0M0 stage. As a result, these patients were either observed after surgery or initially treated with platinum-containing chemotherapy. Additionally, the low rate of PD-L1 expression in our dMMR UC cases further limits the clinical application of ICIs. Thus, a larger cohort of patients receiving ICIs therapy is needed to identify significant biomarkers for immunotherapies. Another potential limitation is that we used a targeted sequencing approach focusing on genetic changes of dMMR UC. A broader sequencing approach, such as whole-exome sequencing, may have identified additional genes with differential alterations in dMMR UC.

## Conclusion

In conclusion, our study reveals the clinicopathological characteristics, genomic landscape, and immunotherapy biomarker landscape of dMMR UC. These unique clinicopathological features contribute to the identification and diagnosis of these tumors. We confirmed the high enrichment of *KMT2D* mutations and *FGFR3* R248C hotspot mutation in dMMR UC. We observed a strong concordance between dMMR and the detection of MSI-H using Promega Analysis System, although NGS showed a lower detection rate of MSI-H. However, we found a correlation between MSI-NGS and TMB and TNB. Furthermore, the density of intratumoral CD8+ T cells may be associated with the therapeutic response to ICIs. Nevertheless, we did not find a correlation between PD-L1 expression and dMMR, MSI-H, TMB, or TNB. Identifying specific molecular and histological biomarkers predictive of immunotherapy response remains a challenge in UC, and further efforts are needed to standardize PD-L1 assessment in UC while evaluating this biomarker in conjunction with other promising but less explored predictors.

## Data availability statement

The datasets presented in this study can be found in online repositories. The names of the repository/repositories and accession number(s) can be found in the article/**Supplementary Material**.

## Ethics statement

The studies involving humans were approved by The internal review and ethics boards of the Shandong Provincial Hospital. The studies were conducted in accordance with the local legislation and institutional requirements. The participants provided their written informed consent to participate in this study.

## Author contributions

YM: Data curation, Writing – original draft. FH: Conceptualization, Writing – review & editing. XZ: Methodology, Writing – review & editing. YX: Data curation, Writing – original draft. JL: Data curation, Methodology, Writing – review & editing. YN: Data curation, Methodology, Writing – original draft. XZ: Data curation, Methodology, Writing – review & editing. JM: Data curation, Methodology, Writing – review & editing. CL: Data curation, Methodology, Writing – review & editing. HZ: Data curation, Methodology, Writing – review & editing. WH: Formal Analysis, Investigation, Writing – review & editing. DS: Formal Analysis, Investigation, Writing – review & editing. MZ: Conceptualization, Supervision, Writing – review & editing. ZY: Conceptualization, Supervision, Writing – original draft, Writing – review & editing.

## Supplementary material

Supplementary Figure 1 Representative microsatellite instability (MSI) gene locus assay using the Promega MSI Analysis System. Compared to the corresponding normal paired sample, Patient 4 exhibited an unstable state at all five microsatellite loci in the tumor sample, indicating MSI-H, while Patient 11 showed stability at all five microsatellite loci, indicating a microsatellite stable (MSS) status.

Supplementary Figure 2 Positive correlations of TMB and TNB with the number of gene mutations **(A, C)**, percentage of unstable MSI loci **(B, D)** in dMMR UC. The positive correlations also presenting between TMB and TNB **(E)**, the density of peritumoral and intratumoral CD8+ T cells **(F)**.

## References

[1] Babjuk M Böhle A Burger M Capoun O Cohen D Compérat EM. EAU guidelines on non-muscle-invasive urothelial carcinoma of the bladder: update 2016. Eur Urol (2017) 71(3):447–61. doi: 10.1016/j.eururo.2016.05.041 27324428

[2] Bellmunt J de Wit R Vaughn DJ Fradet Y Lee JL Fong L. Pembrolizumab as second-line therapy for advanced urothelial carcinoma. N Engl J Med (2017) 376(11):1015–26. doi: 10.1056/NEJMoa1613683 PMC5635424 28212060

[3] Balar AV Castellano D O'Donnell PH Grivas P Vuky J Powles T. First-line pembrolizumab in cisplatin-ineligible patients with locally advanced and unresectable or metastatic urothelial cancer (KEYNOTE-052): a multicentre, single-arm, phase 2 study. Lancet Oncol (2017) 18(11):1483–92. doi: 10.1016/S1470-2045(17)30616-2 28967485

[4] Santopietro AL Einstein D Bellmunt J. Advances in the management of urothelial carcinoma: is immunotherapy the answer? Expert Opin Pharmacother (2021) 22(13):1743–59. doi: 10.1080/14656566.2021.1921149 33905290

[5] Zhu J Armstrong AJ Friedlander TW Kim W Pal SK George DJ. Biomarkers of immunotherapy in urothelial and renal cell carcinoma: PD-L1, tumor mutational burden, and beyond. J Immunother Cancer (2018) 6(1):4. doi: 10.1186/s40425-018-0314-1 29368638 PMC5784676

[6] Yurgelun MB Hampel H. Recent advances in lynch syndrome: diagnosis, treatment, and cancer prevention. Am Soc Clin Oncol Educ Book (2018) 38:101–09. doi: 10.1200/EDBK_208341 30231390

[7] Barrow P Khan M Lalloo F Evans DG Hill J. Systematic review of the impact of registration and screening on colorectal cancer incidence and mortality in familial adenomatous polyposis and Lynch syndrome. Br J Surg (2013) 100(13):1719–31. doi: 10.1002/bjs.9316 24227356

[8] Huang D Matin SF Lawrentschuk N Roupret M. Systematic review: an update on the spectrum of urological Malignancies in lynch syndrome. Bladder Cancer (2018) 4(3):261–68. doi: 10.3233/BLC-180180 PMC6087433 30112437

[9] Audenet F Colin P Yates DR Ouzzane A Pignot G Long JA. A proportion of hereditary upper urinary tract urothelial carcinomas are misclassified as sporadic according to a multi-institutional database analysis: proposal of patient-specific risk identification tool. BJU Int (2012) 110(11 Pt B):E583–9. doi: 10.1111/j.1464-410X.2012.11298.x 22703159

[10] Quintanilha JCF Graf RP Fisher VA Oxnard GR Ellis H Panarelli N. Comparative effectiveness of immune checkpoint inhibitors vs chemotherapy in patients with metastatic colorectal cancer with measures of microsatellite instability, mismatch repair, or tumor mutational burden. JAMA Netw Open (2023) 6(1):e2252244. doi: 10.1001/jamanetworkopen.2022.52244 36689222 PMC9871803

[11] Ma YT Yang HL Yan L Hua F Wang DG Xu GY. Case report: potential predictive value of MMR/MSI status and PD-1 expression in immunotherapy for urothelial carcinoma. Pathol Oncol Res (2022) 28:1610638. doi: 10.3389/pore.2022.1610638 36338826 PMC9633672

[12] Xie Z Liu L Lin X Xie X Gu Y Liu M. A multicenter analysis of genomic profiles and PD-L1 expression of primary lymphoepithelioma-like carcinoma of the lung. Mod Pathol (2020) 33(4):626–38. doi: 10.1038/s41379-019-0391-9 PMC7113185 31659278

[13] Wang M Chen X Dai Y Wu D Liu F Yang Z. Concordance study of a 520-gene next-generation sequencing-based genomic profiling assay of tissue and plasma samples. Mol Diagn Ther (2022) 26(3):309–22. doi: 10.1007/s40291-022-00579-1 35305253

[14] Donahue TF Bagrodia A Audenet F Donoghue MTA Cha EK Sfakianos JP. Genomic characterization of upper-tract urothelial carcinoma in patients with lynch syndrome. JCO Precis Oncol (2018) 2018:PO.17.00143. doi: 10.1200/PO.17.00143 30854504 PMC6404976

[15] Liu M Li N Tang H Chen L Liu X Wang Y. The mutational, prognostic, and therapeutic landscape of neuroendocrine neoplasms. Oncologist (2023) 28(9):e723–36. doi: 10.1093/oncolo/oyad093 PMC10485279 37086484

[16] Hodgson A Vesprini D Liu SK Xu B Downes MR. Correlation of mismatch repair protein deficiency, PD-L1 and CD8 expression in high-grade urothelial carcinoma of the bladder. J Clin Pathol (2020) 73(8):519–22. doi: 10.1136/jclinpath-(2019-206256 31919144

[17] Shang Z Jin S Wang W Wei Y Gu C Yang C. Clinicopathological characteristics and loss of mismatch repair protein expression in Chinese upper tract urothelial carcinomas. Front Oncol (2022) 12:1012168. doi: 10.3389/fonc.2022.1012168 36387191 PMC9640928

[18] Pradere B Lotan Y Roupret M. Lynch syndrome in upper tract urothelial carcinoma: significance, screening, and surveillance. Curr Opin Urol (2017) 27(1):48–55. doi: 10.1097/MOU.0000000000000340 27533503

[19] Goldberg H Wallis CJD Klaassen Z Chandrasekar T Fleshner N Zlotta AR. Lynch syndrome in urologic Malignancies - what does the urologist need to know? Urology (2019) 134:24–31. doi: 10.1016/j.urology.2019.07.004 31302137

[20] Hubosky SG Boman BM Charles S Bibbo M Bagley DH. Ureteroscopic management of upper tract urothelial carcinoma (UTUC) in patients with Lynch Syndrome (hereditary nonpolyposis colorectal cancer syndrome). BJU Int (2013) 112(6):813–9. doi: 10.1111/bju.12008 23452166

[21] Sobrino-Reig E Meizoso T Garcia J Varillas-Delgado D Martin YB. Morphological predictors for microsatellite instability in urothelial carcinoma. Diagn Pathol (2021) 16(1):106. doi: 10.1186/s13000-021-01168-2 34801034 PMC8606048

[22] Urakami S Inoshita N Oka S Miyama Y Nomura S Arai M. Clinicopathological characteristics of patients with upper urinary tract urothelial cancer with loss of immunohistochemical expression of the DNA mismatch repair proteins in universal screening. Int J Urol (2018) 25(2):151–56. doi: 10.1111/iju.13481 29164703

[23] Fujii Y Sato Y Suzuki H Kakiuchi N Yoshizato T Lenis AT. Molecular classification and diagnostics of upper urinary tract urothelial carcinoma. Cancer Cell (2021) 39(6):793–809 e8. doi: 10.1016/j.ccell.2021.05.008 34129823 PMC9110171

[24] Juanpere N Agell L Lorenzo M de Muga S López-Vilaró L Murillo R. Mutations in FGFR3 and PIK3CA, singly or combined with RAS and AKT1, are associated with AKT but not with MAPK pathway activation in urothelial bladder cancer. Hum Pathol (2012) 43(10):1573–82. doi: 10.1016/j.humpath.2011.10.026 22417847

[25] Sharma P Siefker-Radtke AO De Braud F Basso U Calvo E Bono P. Nivolumab (N) alone or in combination with ipilimumab (I) in patients (pts) with platinum-pretreated metastatic urothelial carcinoma (mUC): Extended follow-up from CheckMate 032. (2020) 31(4_suppl):S582–3. doi: 10.1016/j.annonc.2020.08.821

[26] Powles T van der Heijden MS Castellano D Galsky MD Loriot Y Petrylak DP. Durvalumab alone and durvalumab plus tremelimumab versus chemotherapy in previously untreated patients with unresectable, locally advanced or metastatic urothelial carcinoma (DANUBE): a randomised, open-label, multicentre, phase 3 trial. Lancet Oncol (2020) 21(12):1574–88. doi: 10.1016/S1470-2045(20)30541-6 32971005

[27] Glaser AP Fantini D Shilatifard A Schaeffer EM Meeks JJ. The evolving genomic landscape of urothelial carcinoma. Nat Rev Urol (2017) 14(4):215–29. doi: 10.1038/nrurol.2017.11 28169993

[28] Wang P Chen Y Wang C. Beyond tumor mutation burden: tumor neoantigen burden as a biomarker for immunotherapy and other types of therapy. Front Oncol (2021) 11:672677. doi: 10.3389/fonc.2021.672677 33996601 PMC8117238

[29] Powles T Kockx M Rodriguez-Vida A Duran I Crabb SJ Van Der Heijden MS. Clinical efficacy and biomarker analysis of neoadjuvant atezolizumab in operable urothelial carcinoma in the ABACUS trial. Nat Med (2019) 25(11):1706–14. doi: 10.1038/s41591-019-0628-7 31686036

[30] Jia Y Liu L Shan B. Future of immune checkpoint inhibitors: focus on tumor immune microenvironment. Ann Transl Med (2020) 8(17):1095. doi: 10.21037/atm-20-3735 33145314 PMC7575936

[31] Yang Y Jain RK Glenn ST Xu B Singh PK Wei L. Complete response to anti-PD-L1 antibody in a metastatic bladder cancer associated with novel MSH4 mutation and microsatellite instability. J Immunother Cancer (2020) 8(1):e000128. doi: 10.1136/jitc-2019-000128 32221012 PMC7206971

[32] Chen S Zhang N Wang T Zhang E Wang X Zheng J. Biomarkers of the response to immune checkpoint inhibitors in metastatic urothelial carcinoma. Front Immunol (2020) 11:1900. doi: 10.3389/fimmu.2020.01900 32983112 PMC7477044

[33] Faraj SF Munari E Guner G Taube J Anders R Hicks J. Assessment of tumoral PD-L1 expression and intratumoral CD8+ T cells in urothelial carcinoma. Urology (2015) 85(3):703 e1–6. doi: 10.1016/j.urology.2014.10.020 PMC4695997 25733301

[34] Dason S McPherson V Teo MY Isharwal S Audenet F Bagrodia A. Defining the DNA damage repair (DDR) genomic landscape of urothelial carcinoma of the bladder (UCB). J Clin Oncol (2018) 36:502–02. doi: 10.1200/JCO.2018.36.6_suppl.502

[35] Vidotto T Nersesian S Graham C Siemens DR Koti M. DNA damage repair gene mutations and their association with tumor immune regulatory gene expression in muscle invasive bladder cancer subtypes. J Immunother Cancer (2019) 7(1):148. doi: 10.1186/s40425-019-0619-8 31174611 PMC6556053

