# Supplementary figures and images for: Clinicopathological characteristics, molecular landscape, and biomarker landscape for predicting the efficacy of PD-1/PD-L1 inhibitors in Chinese population with mismatch repair deficient urothelial carcinoma: a real-world study
Source: Front Immunol. 2023 Nov 6;14:1269097. doi: 10.3389/fimmu.2023.1269097 (PMC10657814; doi:10.3389/fimmu.2023.1269097)

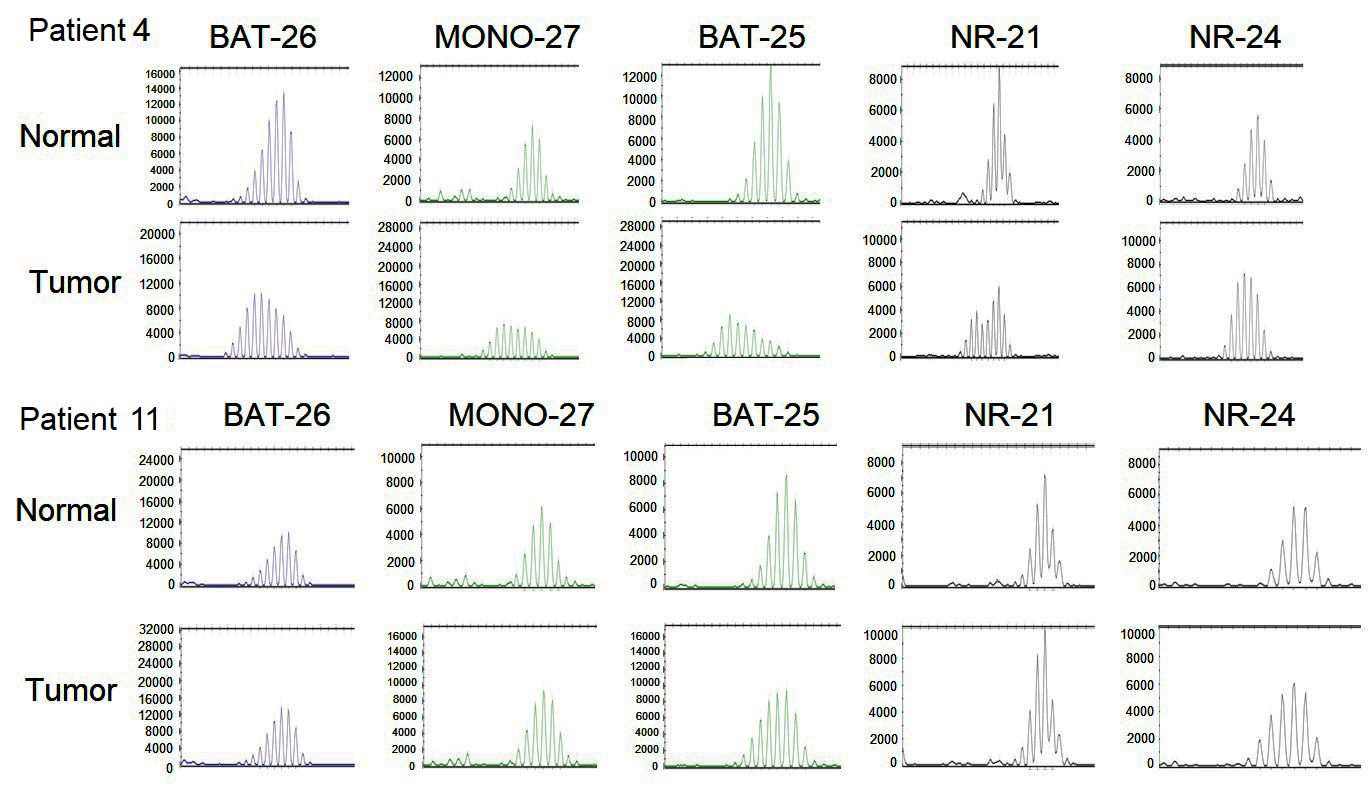

Supplement: Supplementary Figure 1 — Representative microsatellite instability (MSI) gene locus assay using the Promega MSI Analysis System. Compared to the corresponding normal paired sample, Patient 4 exhibited an unstable state at all five microsatellite loci in the tumor sample, indicating MSI-H, while Patient 11 showed stability at all five microsatellite loci, indicating a microsatellite stable (MSS) status. [file Image_1.jpeg]

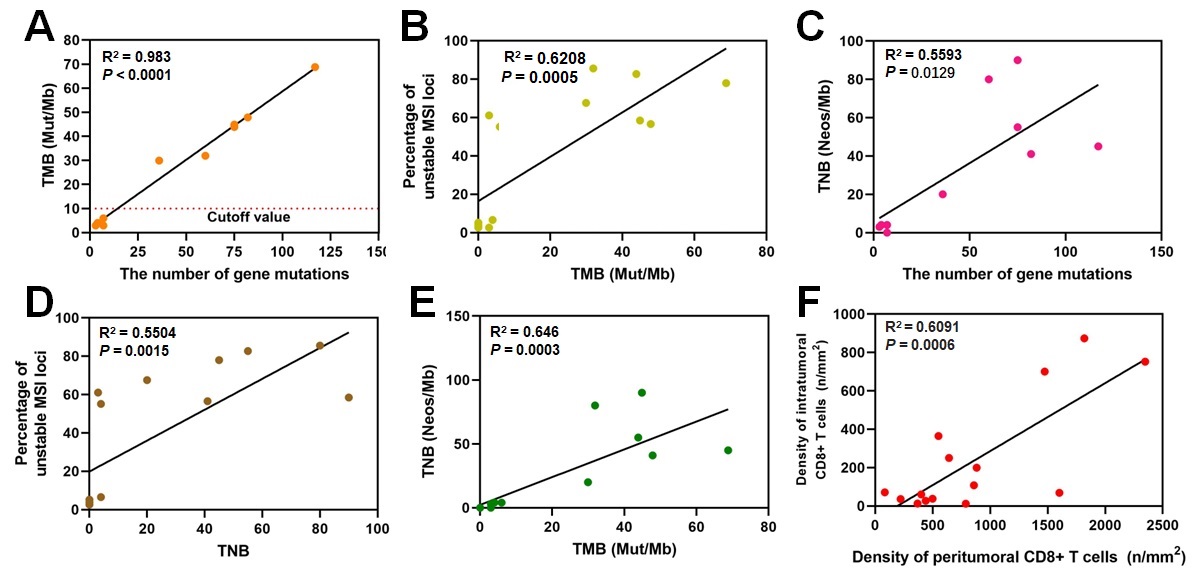

Supplement: Supplementary Figure 2 — Positive correlations of TMB and TNB with the number of gene mutations (A, C), percentage of unstable MSI loci (B, D) in dMMR UC. The positive correlations also presenting between TMB and TNB (E), the density of peritumoral and intratumoral CD8+ T cells (F). [file Image_2.jpeg]
