# Supplementary material for: Clinicopathological characteristics, molecular landscape, and biomarker landscape for predicting the efficacy of PD-1/PD-L1 inhibitors in Chinese population with mismatch repair deficient urothelial carcinoma: a real-world study
Source: Front Immunol. 2023 Nov 6;14:1269097. doi: 10.3389/fimmu.2023.1269097 (PMC10657814; doi:10.3389/fimmu.2023.1269097)
Supplement: Supplementary file 3 [file Table_1.docx]

Supplementary table 1. Biomarkers to predict the efficacy of ICIs therapy in patients with dMMR UC.

| Patient | Instable MSI loci (%) | MSI-NGS | MSI-PCR | TMB (Mut/Mb)* | TNB (Neos/Mb)** | PD-1 (CPS) | PD-L1 (CPS) | PD-L1 (TPS, %) |
| --- | --- | --- | --- | --- | --- | --- | --- | --- |
| 1 | 58.44 | High | High | 44.87 | 90 | 60 | 5 | 5 |
| 2 | 2.6 | MSS | High | 2.99 | 0 | 0 | 15 | 10 |
| 3 | 3.85 | MSS | High | 0 | 0 | 5 | 0 | 0 |
| 4 | 2.6 | MSS | High | 0 | 0 | 5 | 0 | 0 |
| 5 | 5.19 | MSS | High | 0 | 0 | 5 | 0 | 0 |
| 6 | 61.04 | High | High | 2.99 | 3 | 0 | 0 | 0 |
| 7 | 67.53 | High | ND | 29.91 | 20 | 0 | 0 | 0 |
| 8 | 85.53 | High | ND | 31.9 | 80 | 0 | 0 | 0 |
| 9 | 77.92 | High | High | 68.79 | 45 | 0 | 0 | 0 |
| 10 | 56.58 | High | High | 47.86 | 41 | 0 | 0 | 0 |
| 11 | 3.9 | MSS | Low | 0 | 0 | 1 | 0 | 0 |
| 12 | 6.58 | MSS | High | 3.99 | 4 | 1 | 0 | 0 |
| 13 | 3.85 | MSS | High | 0 | 0 | 5 | 0 | 0 |
| 14 | 55.13 | High | ND | 5.98 | 4 | 10 | 5 | 1 |
| 15 | 82.67 | High | High | 43.87 | 55 | 8 | 0 | 0 |

*: TMB > 10 Muts/Mb was defined as TMB-High.

**: TNB > 4.5 Neos/Mb was defined as TNB-High.
