# Supplementary material for: Clinicopathological characteristics, molecular landscape, and biomarker landscape for predicting the efficacy of PD-1/PD-L1 inhibitors in Chinese population with mismatch repair deficient urothelial carcinoma: a real-world study
Source: Front Immunol. 2023 Nov 6;14:1269097. doi: 10.3389/fimmu.2023.1269097 (PMC10657814; doi:10.3389/fimmu.2023.1269097)
Supplement: Supplementary file 4 [file Table_2.docx]

Supplemental table 2. Evaluation of T lymphocytes in the microenvironment of UC with dMMR

| Patient | Density of peritumoral CD3+ T cell (n/mm^2^) | Density of peritumoral CD8+ T cell (n/mm^2^) | Density of intratumoral CD8 + T cells (n/mm^2^) | Peritumoral CD8/CD3 (%) | Peritumoral CD4/CD3 (%) |
| --- | --- | --- | --- | --- | --- |
| 1 | 4070.54 | 1819.00 | 873.60 | 46.61 | 48.29 |
| 2 | 3566.21 | 2347.27 | 752.22 | 65.05 | 14.19 |
| 3 | 3508.08 | 882.15 | 199.34 | 26.65 | 7.48 |
| 4 | 3770.50 | 1602.23 | 68.51 | 43.31 | 52.85 |
| 5 | 4030.02 | 400.56 | 60.45 | 10.58 | 72.79 |
| 6 | 2248.11 | 499.20 | 37.61 | 24.79 | 52.17 |
| 7 | 3882.29 | 858.78 | 107.70 | 23.25 | 63.39 |
| 8 | 2179.56 | 440.05 | 27.48 | 19.68 | 8.40 |
| 9 | 1656.76 | 221.90 | 35.90 | 13.42 | 16.65 |
| 10 | 724.01 | 84.62 | 71.19 | 11.11 | 8.33 |
| 11 | 2931.95 | 642.81 | 250.26 | 20.58 | 15.22 |
| 12 | 2212.78 | 787.74 | 12.09 | 37.17 | 53.30 |
| 13 | 1713.65 | 369.06 | 13.02 | 22.76 | 56.38 |
| 14 | 2647.81 | 551.00 | 364.83 | 21.44 | 18.57 |
| 15 | 3483.53 | 1474.92 | 700.52 | 42.34 | 27.03 |
